# Sol–Gel Entrapped Lewis Acids as Catalysts for Biodiesel Production

**DOI:** 10.3390/molecules25245936

**Published:** 2020-12-15

**Authors:** Mirit Kolet, Melad Atrash, Karen Molina, Daniel Zerbib, Yael Albo, Faina Nakonechny, Marina Nisnevitch

**Affiliations:** Department of Chemical Engineering, Ariel University, Kyriat-ha-Mada, Ariel 4070000, Israel; miritk@ariel.ac.il (M.K.); melada@ariel.ac.il (M.A.); karenm@ariel.ac.il (K.M.); daniz1234@gmail.com (D.Z.); yaelyt@ariel.ac.il (Y.A.); fainan@ariel.ac.il (F.N.)

**Keywords:** Lewis acids, sol–gel, biodiesel, free fatty acids

## Abstract

Replacing fossil fuels with biodiesel enables the emission of greenhouse gases to be decreased and reduces dependence on fossil fuels in countries with poor natural resources. Biodiesel can be produced by an esterification reaction between free fatty acids (FFAs) and methanol or by transesterification of triglycerides from oils. Both reactions require homogeneous or heterogeneous catalysis. Production of biodiesel catalyzed by heterogeneous catalysts seems to be the preferred route, enabling easy product separation. As we have previously shown, the Lewis acids AlCl_3_ and BF_3_ can serve as highly efficient catalysts under ultrasonic activation. The present study focused on the development of oleic acid (OA) esterification with methanol by the same catalysts immobilized in silica matrices using the sol–gel synthesis route. During the course of immobilization, AlCl_3_ converts to AlCl_3_ × 6H_2_O (aluminite) and BF_3_ is hydrolyzed with the production of B_2_O_3_. The immobilized catalysts can be reused or involved in a continuous process. The possibility of biodiesel production using immobilized catalysts under ultrasonic activation is shown for the conversion of FFAs into biodiesel in batch and continuous mode.

## 1. Introduction

Technological and demographic changes have caused an increase in the consumption of energy originating mainly from the combustion of fossil fuels, a nonrenewable natural resource, which has resulted in growing emissions of greenhouse gases [1,2]. Today, global warming and climate change can be considered as proven facts and are recognized as complex problems that do not have a single clear solution. The increased energy consumption has led to a search for alternative energy sources and prompted studies on renewable energy [3]. One of the most prominent economic alternatives to fossil oil is biodiesel, which is usually produced from the biomass of various crops. However, an increased use of crops for fuel production may lead to dilution of the world’s food sources [4,5]. The use of alternative sources for biodiesel production is therefore being studied extensively [6]. World biodiesel production increased by 70% between 2005 and 2015 and is predicted to rise by another 35% by 2025. For these reasons, there is a keen interest in developing efficient and cost-effective biodiesel production processes based on sustainable sources [2,7].

Biodiesel is a mixture of alkyl esters of fatty acids produced by free fatty acid (FFA) esterification or triglyceride transesterification with alcohol in the presence of catalysts. These two reactions require activation, which is usually obtained by heating the reaction mixture to high temperatures [8,9]. Ultrasonication has recently been proposed as an alternative method for activation of these reactions [10,11,12].

The catalysts used for accelerating the esterification and transesterification reactions are either chemical or biological. The biological catalysts are enzymes. These enzymes tolerate high FFA and water content in the reaction mixture during the conversion of fatty acids or triglycerides into biodiesel without soap formation. The biodiesel yield is relatively high under moderate operating conditions, and it is easy to separate the enzymes when they are used in a heterogeneous form. The disadvantages of enzymatic catalysis include difficulties in large-scale application and the high overall cost of biodiesel production, together with a slow reaction rate [13].

Chemical catalysts are acids and bases that are used in heterogeneous and homogeneous forms [13]. The application of homogeneous and heterogeneous acid catalysts requires higher process temperatures compared to homogeneous or heterogeneous base catalysts [13,14]. The activity of base catalysts is higher than that of acid catalysts for both homogeneous and heterogeneous catalysts, and the reaction occurs under milder conditions. However, base catalysts cause the problem of a side saponification reaction that occurs in the presence of FFA [15]. The heterogeneous base and acid catalysts are much easier to separate from the product, and allow the performance of continuous processes or reuse of the catalysts in batch regimes [16,17]. Solid base catalysts, such as CaO, have advantages over homogeneous ones since they exhibit higher catalytic activity and have longer lifetimes. The drawback of solid base catalysts is a longer reaction time than in the case of homogeneous catalysts. The transesterification of waste cooking oil under base heterogeneous catalysts using methanol is usually performed under ambient pressure at temperatures around 65°C, since above this temperature methanol evaporates, and as a consequence, the reaction yield decreases [18]. Contrary to base catalysts, heterogeneous and homogeneous acid catalysts do not cause any side reactions with FFAs and can therefore be used in both esterification and transesterification reactions [19].

Lewis acids are effective catalysts for esterification reactions in both homogeneous and heterogeneous forms [20,21] and are active under mild conditions [22,23]. One of the advantages of using Lewis acids is reduced corrosion of the reaction facilities, especially when they are used in the heterogeneous form [24]. In our previous publications we showed that BF_3_ and AlCl_3_ are effective catalysts in the esterification reaction between oleic acid (OA) and methanol [12] and in biodiesel production using brown grease as a source of FFA [25]. A 100% yield was achieved under ultrasonic activation and a 15 min reaction time in the reaction between OA and methanol when using brown grease [12,25]. To develop a continuous process for biodiesel production, it was decided to incorporate BF_3_ and AlCl_3_ into silica matrices by a versatile sol–gel synthesis route. The main challenge in using heterogeneous catalysts is overcoming the limits of diffusion, thus allowing higher yields [18,26]. Immobilization of the catalyst will enable a longer reaction response cycle and the development of an efficient continuous process.

The aim of the present study was to determine whether a Lewis acid (BF_3_ and AlCl_3_) could be immobilized in silica matrices through a sol–gel process in order to obtain effective catalysts that can be reused in alternating reaction cycles. An additional goal was to develop a method for producing biodiesel using immobilized catalysts that will remain active for a long time.

## 2. Results and Discussion

### 2.1. Catalyst Characterization

Two catalysts were incorporated into silica matrices by the sol–gel synthesis route. Two matrices were obtained by the entrapment of BF_3_ and AlCl_3_ in the sol–gel network (designated BSil and AlSil, respectively). A blank matrix, prepared without a catalyst (designated BlankSil), was used in the control experiments. HCl was used as an acid catalyst when preparing sol–gel matrices by the hydrolytic polycondensation of the tetraehthylorthosilane precursor. Use of base catalysts was avoided at this stage to ensure acidic reaction conditions in the further esterification process. Sol–gel matrices prepared via the one step procedure under acid catalysis usually have pore size in the micropore range [27]. The prepared matrices were characterized by Fourier transform infrared (FTIR), X-ray diffraction (XRD) and a particle sizing counter.

**Figure 1:**
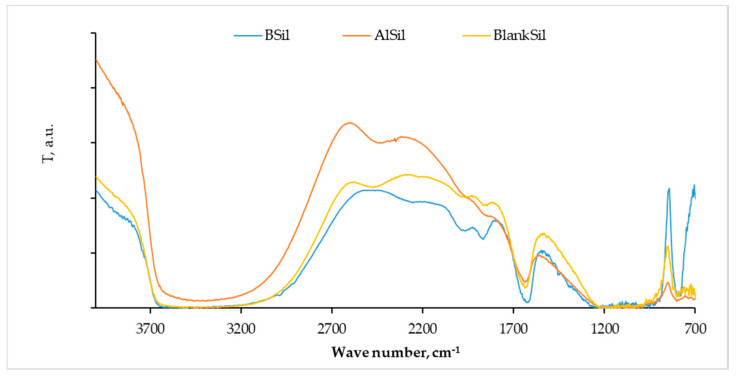
Fourier transform infrared (FTIR) spectra of the blank matrix and matrices with the immobilized catalysts BF_3_ and AlCl_3_. BSil—blue, AlSil—orange, BlankSil—yellow.

Identification of the functional groups in the prepared matrices was performed by FTIR spectroscopy (Figure 1). The spectra contained bands of functional groups characteristic of silica matrices prepared by the sol–gel technique. The broad band at 3500 cm^−1^ could be assigned to the stretching vibrations of -OH groups, and the intense peak at 1610 cm^−1^ could be related to H-O-H deformation indicating the presence of water and residual hydroxyl groups in the matrices, implying the hydrophilic character of the latter. The bond of asymmetric stretching frequency of Si-O-Si was broad, with maximum absorbance around 1100 cm^−1^.

Since all characteristic bands of the BlankSil were also found in the spectra of the catalyst-incorporated matrices, we assumed that the entrapment of BSil or AlSil did not alter the internal structure of the matrix.

**Figure 2:**
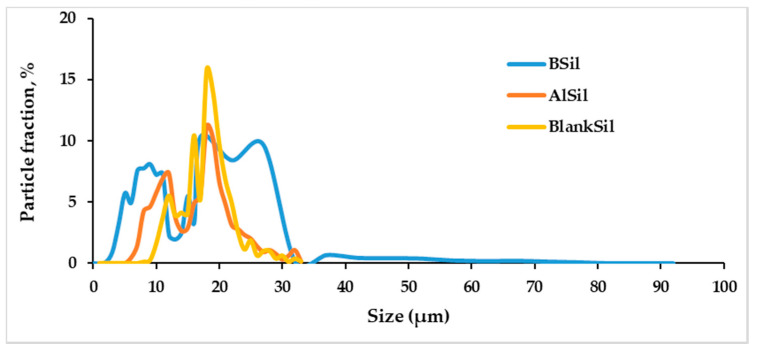
Distribution of the particle size of sol–gel matrix powders of BlankSil, BSil and AlSil.

The particle size of the sol–gel powders was measured using a laser particle counter. Figure 2 shows the particle size distribution of the three matrices: BSil, AlSil and BlanSil. The particle sizes ranged between 2 and 92 μm, i.e., the samples were far from being homogeneous in size. The particle size of BlankSil was 23.05 ± 3.9 μm, BSil was 14.06 ± 3.03 μm and AlSil was 22.05 ± 2.9 μm. The observed heterogeneity can be explained by the procedure for preparation of the sol–gel matrices that were manually crushed into a powder with a mortar and pestle after the drying stage.

**Figure 3:**
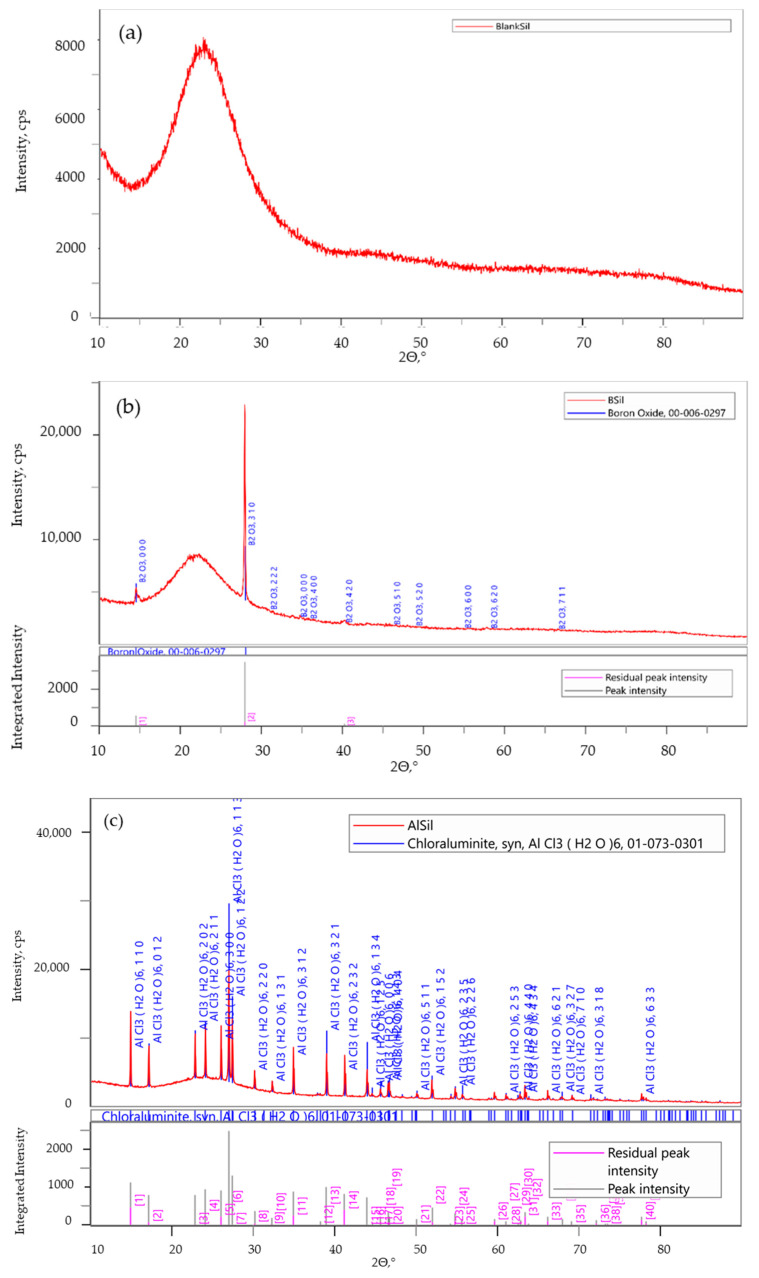
X-ray diffraction (XRD) analysis of the immobilized catalysts (**a**) BlankSil; (**b**) BSil and (**c**) AlSil.

XRD analysis was performed in order to gain an insight into the catalyst phases entrapped in the matrices. The diffraction patterns obtained for the three matrices are shown in Figure 3. The broad diffraction peak at 20° = 23° that is present in all spectra is attributed to the amorphous silica host matrix, as demonstrated in Figure 3a.

Figure 3b shows that as per the XRD pattern, the BSil matrix contains boron oxide, rather than BF_3_ (Figure 3b). BF_3_ was probably hydrolyzed to boric acid in the course of the sol–gel formation in the aqueous medium [28,29], which in turn was converted into boron oxide during the sol–gel process. Since boron oxide is a Lewis acid [29] that can be used as a catalyst in esterification reactions, e.g.**,** in the studies of Carlson et al. [30] and Murakami et al. [31], it was decided to examine the catalytic performance of this catalyst. The results of the XRD analysis of the AlSil matrix confirm the presence of AlCl_3_, but in the form of its hydrate, AlCl_3_×6H_2_O (chloraluminite) (Figure 3c).

### 2.2. Catalytic Tests

**Scheme 1:**
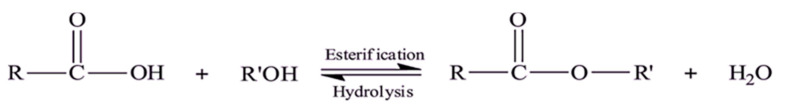
Esterification reaction of FFA with alcohol.

The prepared matrices were tested as catalysts in the esterification reaction of OA with methanol Scheme 1. In this reaction, a nucleophilic attack of alcohol occurs after protonation of a carbonyl group, resulting in an intermediate tetrahedral product, which subsequently converts into an ester [12,32].

Since the presence of water reduces the reaction yields, all matrices were dried in a vacuum oven prior to use. In addition, the reactions were carried out using a high molar excess of methanol (1:176) in the presence of the three matrices under ultrasonic activation at the ambient temperature, first in a batch regime and then in a continuous mode. Monitoring of the sample composition during the course of the reaction was performed by HPLC.

**Figure 4:**
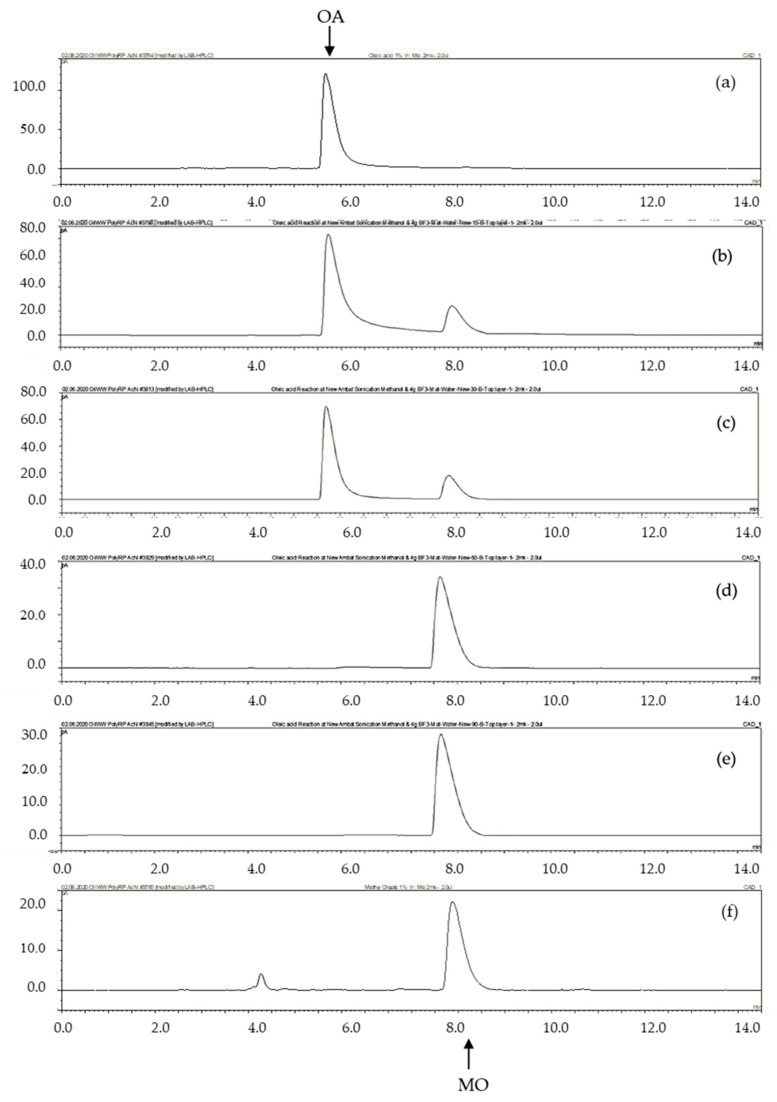
Esterification of oleic acid (OA) by methanol under ultrasonic activation using the BSil matrix as a catalyst. HPLC chromatograms of OA (**a**) before esterification, and after esterification: (**b**) after 15 min, (**c**) 30 min, (**d**) 60 min and (**e**) 90 min. (**f**) A commercial standard of methyl oleate (MO).

Figure 4 shows chromatograms of oleic acid (OA) before (Figure 4a) and after esterification for several reaction times: 15 min (Figure 4b), 30 min (Figure 4c), 60 min (Figure 4d) and 90 min (Figure 4e) under ultrasonic activation of the reaction catalyzed by the BSil matrix. In all cases, the reaction was followed by phase separation with water, and the product of the reaction, the methyl ester of OA, was found only in the upper phase, whereas the lower phase did not contain any product. The product was identified by matching the retention time to that of the commercial MO (methyl ester of OA) standard (Figure 4f). Similar chromatograms were obtained for the other matrices (data not shown).

The yield of the product of esterification was calculated as the percent of the MO peak area relative to the sum of the areas of OA and MO peaks from the chromatograms in Figure 4 for BSil or similar chromatograms for AlSil and BlankSil.

**Figure 5:**
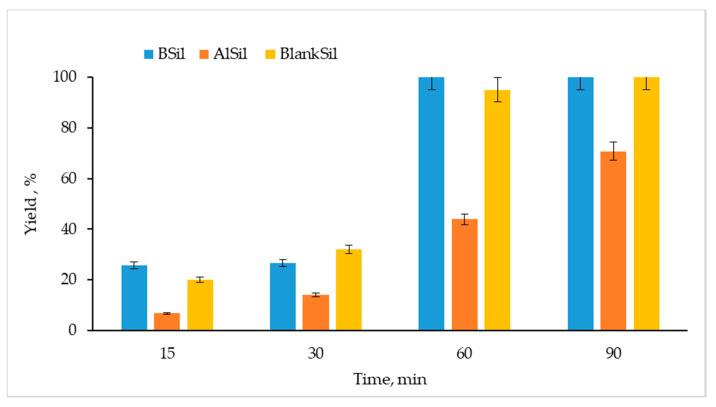
Efficiencies of OA esterification with methanol catalyzed by the immobilized catalysts (BSil, AlSil, and BlankSil) under ultrasonic activation for 15–90 min.

Figure 5 shows results of esterification performed using the three catalysts at different durations of ultrasonic activation. After 15 and 30 min of activation, the obtained yields were lower than 30% for all catalysts. After 60 min of OA esterification using BSil, a 100% yield was obtained. For the other matrices, BlankSil and AlSil, the yields were 95 and 44%, respectively. After 90 min of activation, a yield of 100% was achieved in the reaction using BlankSil, whereas the yield was only 70% under AlSil catalysis. This moderate AlSil activity can be explained by the presence of water molecules bound to AlCl_3_ in chloraluminite. The catalytic activity of the BlankSil matrix can be attributed to its acidic nature.

Immobilization of the catalysts led to a certain decrease in their catalytic performance compared to free catalysts. In our previous study, OA esterification was performed for 15 min under ultrasonic activation using the free catalyst AlCl_3_, gaining a 100% yield [12], whereas only a 7% yield was obtained using the immobilized catalyst AlSil in the same reaction for the same length of time (Figure 5). This phenomenon can probably be explained not only by poor availability of the reagents to AlCl_3_ in the matrix, but also by a hydrated form of AlCl_3_ in chloraluminite. A reduction in the reaction yield was also observed for the catalyst BF_3_: a 100% yield was obtained for the free catalyst after 15 min of reaction catalyzed by BSil, compared to a 25% yield when using the immobilized catalyst under the same reaction conditions. At the same time, it should be mentioned that direct comparison between the free and immobilized BF_3_ is problematic, since BF_3_ was converted into boron oxide during the course of immobilization. However, a 100% yield of esterification can be achieved within reasonable time periods under ambient conditions, opening prospects for continuous production of biodiesel. Even in the case of AlCl_3_, which in its free form is considered a heterogeneous catalyst, immobilizing it into a sol–gel matrix makes sense, since AlCl_3_ possesses limited solubility in methanol [33] and may leak from the continuous reactor when applied in its free form.

Fawaz et al. [34], who examined esterification of linoleic acid (LA) with methanol catalyzed by HZSM-5 (zeolite crystals of distinct Si/Al), obtained a yield of only 79.78% after 4 h of reaction at 180 °C. In our study, esterification of OA with methanol using the BSil matrix led to a 100% yield after 1 h of ultrasonic activation. We assume that the observed difference in yields can be assigned to the type of activation and not to the source of the fatty acid. In our previous work [12] we demonstrated that there was no difference in reaction yield between esterification of LA and OA under the same conditions. Since thermal activation of reactions is an energy-consuming process, and in addition some time is required for heating the reactant mixture to the reaction temperature, ultrasonic activation could become an alternative way of performing reactions while saving energy and time [12].

Ultrasonic activation seems to have advantages over thermal activation not only in the case esterification, but also in transesterification reactions. Ma et al. reported a 92% yield of waste cooking oil transesterification with methanol using FeCl_3_ as a heterogeneous catalyst performed at 90 °C for 120 min [35]. Transesterification of canola oil with methanol using Li/TiO_2_ as a heterogeneous catalyst at 55 °C for 3 h resulted in a 98% yield as reported by Alsharifi et al. [36]. Gardy et al. [37] produced biodiesel with a 98.3% yield using a mesoporous TiO_2_/PrSO_3_H solid acid catalyst (4.5%, *w*/*w*) by the transesterification of used cooking oil at 60 °C for 9 h. Abukhadra et al. [38] performed transesterification of palm oil with methanol in the presence of muscovite/synthetic zeolite (phillipsite) (Mu/Pz) using mechanical stirring. A 94% yield of biodiesel was obtained after 180 min at 100°C and 1300 rpm. In that study, a 97.8% yield was achieved after 90 min for the same reaction mixture, but under ultrasonic activation [38].

For using the obtained catalysts in a continuous process, we examined the reusability of the matrices by performing eight successive OA esterification experiments under ultrasonic activation.

**Figure 6:**
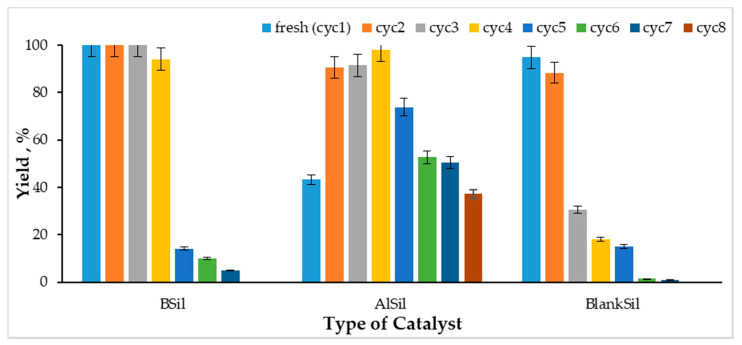
Efficiency of the recycled catalysts BSil, AlSil and BlankSik in OA esterification with methanol under ultrasonic activation for 60 min.

The efficiency of the esterification reaction was tested after eight cycles of catalyst use. As shown in Figure 6, BSil maintained its high ability to catalyze the reaction for four cycles, providing almost 100% yield of the product, while BlankSil was highly active only in two cycles. Surprisingly, the AlSil matrix was active for all eight cycles, where the reaction efficiency increased with repeated use. Ultrasonic processing probably caused grinding of the sol–gel powder particles, thus increasing the surface area of the catalyst and providing better contact between the reagents and the catalyst.

The possibility of reusing catalysts was discussed in the works of several groups. Negm et al. [39] reported OA esterification with methanol using new heterogeneous catalysts, in which phosphotungstic acid was chemically bonded to hydrophobic highly crosslinked poly(4-divinylbenzene-co-vinylbenzyl chloride) via a soft linker, ethylenediamine or 1.8-diaminooctane. Both catalysts were recycled seven times, and the yields of catalyzed reactions were around 98% in all cycles. Dai et al. [40] studied the reusability of the catalyst LiFe_5_O_8_-LiFeO_2_ in transesterification of soybean oil with methanol. This catalyst was found to preserve its activity for 5 runs, providing a reaction yield of 94%. The catalysts used in both works [39,40] were found to be more reusable than those proposed in the present study, but it must be noted that in those studies the reactions were carried out under thermal activation for twice as long.

We assumed that the sol–gel based catalysts still could be considered as prospective for continuous use, which was studied in the next step of our work.

**Figure 7:**
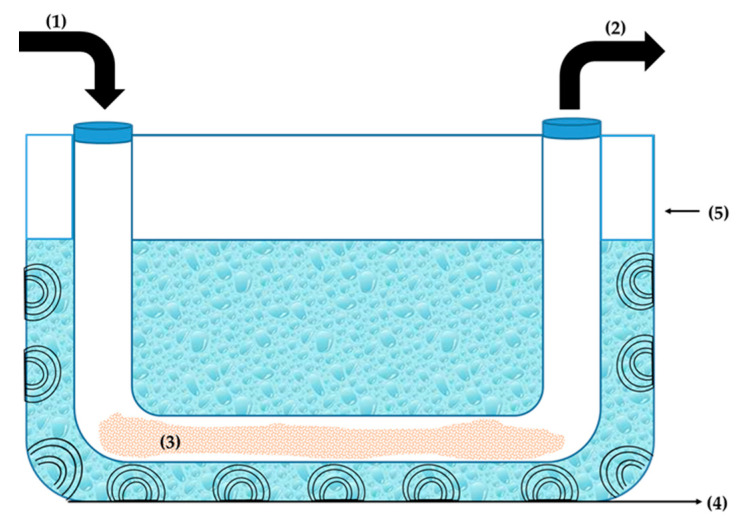
Scheme of the experimental setup for continuous biodiesel production under ultrasonic activation. (1)—Inlet; (2)—outlet; (3)—matrix; (4)—ultrasonic transducer; (5)—ultrasonic bath.

**Figure 8:**
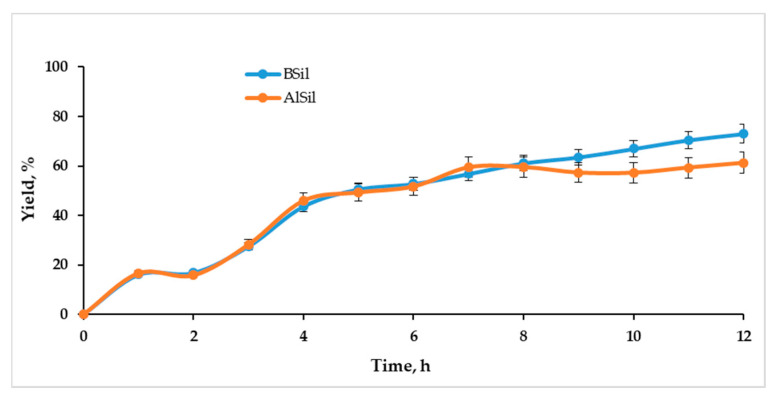
Efficiency of continuous OA esterification with methanol catalyzed by BSil and AlSil under ultrasonic activation.

The performance of the catalysts BSil and AlSil in a continuous mode was tested using a fixed-bed flow reactor. The scheme of the experimental apparatus is shown in Figure 7. The mixture of reagents flowed through a glass tube filled with matrices and the samples collected at the outlet were analyzed by HPLC. The results of the experiments are presented in Figure 8. At the initial period of the experiment, when the reactor operated in a transition state, the product yield was very low, since before the experiment the reactor contained only solvents, and the product at the outlet was very diluted. After 7–9 h in both cases, the process reached a stationary state and the efficiency of the reaction reached satisfactory yields of 58.8 ± 1.9% for AlSil and 68.6 ± 4.1 for BSil, where the advantage of BSil was statistically confirmed by a *p*-value of 0.006. The experiment results proved a principal possibility of continuous biodiesel production under ultrasonic activation using sol–gel immobilized catalysts.

Suranani et al. [41] studied continuous high-yield transesterification of cooking oils catalyzed by sulfuric acid under thermal activation at 80 °C in an Advanced Flow Reactor. However, application of a corrosive homogeneous catalyst may be very problematic for product separation and maintenance of equipment, whereas inert solid-phase catalysts lack these drawbacks.

**Figure 9:**
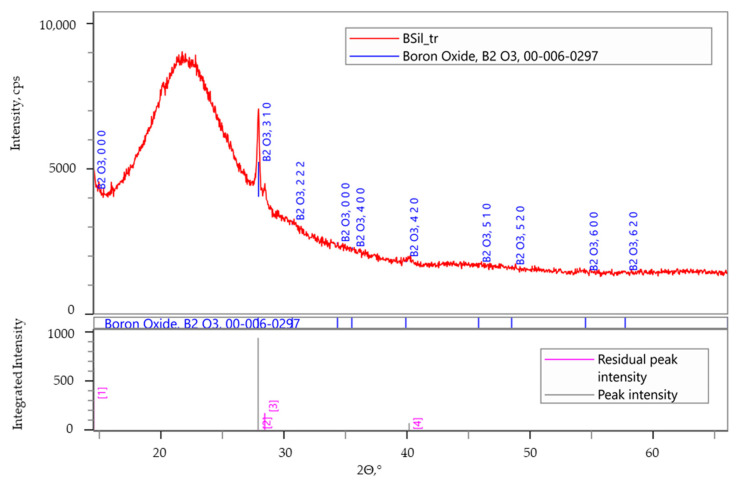
XRD analysis of the used immobilized catalyst BSil.

Since the catalytic performance of the BSil matrix was the highest in both batch and continuous modes, it was decided to characterize this matrix after use by XRD and X-ray photoelectron spectroscopy (XPS). Figure 9 presents the XRD pattern of the used BSil, which was very close to that of a freshly prepared matrix (Figure 3b), showing the presence of the boron-based catalyst within the matrix of the used catalyst.

**Figure 10:**
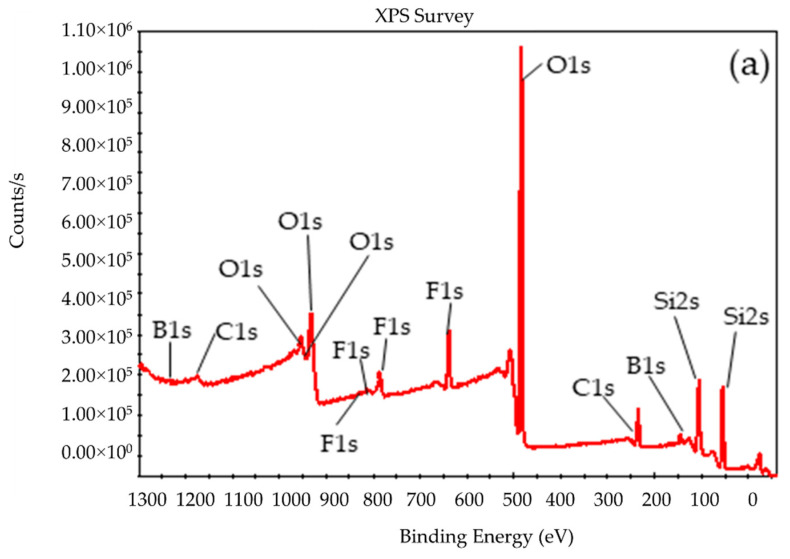

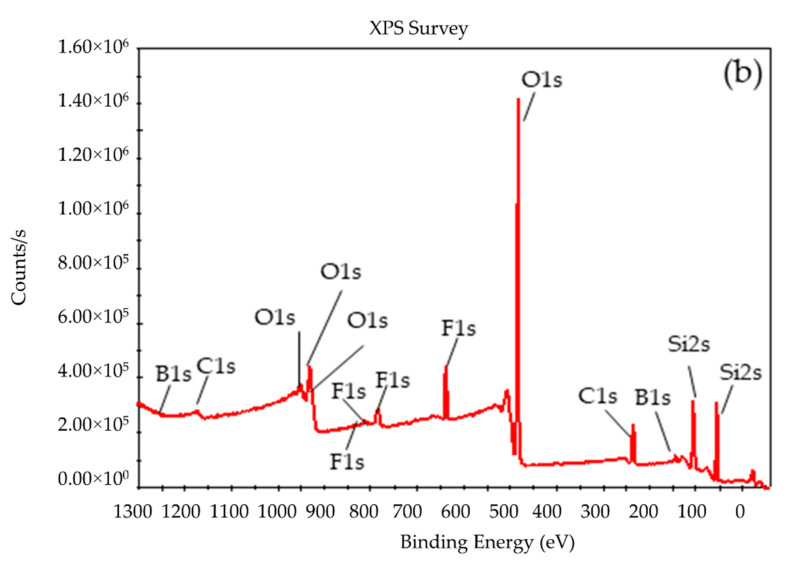
X-ray photoelectron spectroscopy (XPS) analysis of BSil (**a**) before and (**b**) after use in the reaction of biodiesel production.

To compare the composition of the BSil matrix before and after use, XPS measurements were performed. Figure 10 presents the XPS spectra of the freshly prepared (Figure 10a) and the used (Figure 10b) BSil matrices, and Table 1 summarizes their composition. It can be noticed that the XPS spectra in both cases were very close. Data presented in Table 1 showed that the difference in the composition of the matrices was very small. The results of XRD and XPS indicated the stability of the BSil matrix during the catalytic experiments.

## 3. Materials and Methods

### 3.1. Catalyst Preparation

#### 3.1.1. Preparation of BSil

A mixture of 8.0 mL H_2_O (1.365 g, 0.076 mol) and 0.185 mL HCl 37% (Merck, Germany) (0.037 g, 0.38 mmol) was added dropwise under continuous stirring to a solution containing 25 mL (2.77 g, 0.019 mol) tetraethoxysilane (TEOS) (Merck, Germany) dissolved in 26 mL (0.776 g, 0.019 mol) ethanol (Bio Lab., Ashkelon, Israel). The mixture was stirred for 10 min, after which 0.028 mol BF_3_ (Merck, Germany) dissolved in 8.64 mL methanol were added. The solution was stirred for 20 min. The reaction beaker was then covered with parafilm that was punched with a needle and left for gelation, aging and drying for 14 days at room temperature. The dry gel was crushed with a mortar and pestle into a powder and then kept in a vacuum oven (Shel Lab., Cornelius, OR, USA, SVAC1) at 80 °C for 3 h, to ensure complete dryness of the catalyst before use in the esterification processes (Section 3.3 and Section 3.4).

#### 3.1.2. Preparation of AlSil

The AlCl_3_ catalyst was immobilized by the same procedure, except for the addition 0.028 mol of AlCl_3_ (Fluorochem, United Kingdom) dissolved in 20 mL of ethanol, which was carried out after one week of gelation to prevent reaction of ACl_3_ with water.

#### 3.1.3. Preparation of BlankSil

Blank sol–gel was prepared by the same procedure without the addition of any Lewis acids.

### 3.2. Characterization of Prepared Matrices

#### 3.2.1. FTIR analysis of the immobilized catalysts

FTIR measurements were performed using a Perkin Elmer Spectrum One spectrometer (Perkin Elmer, Waltham, MA). The spectra were measured using transmittance mode in KBr (Merck, Germany) pellets containing 1% in weight powder samples. The pellets were prepared using a press (Equilab, Madrid, Spain) under 5000 kg_f_ for 1 h with the help of a 13 mm pellet die (Carver, Wabash, IN, USA).

#### 3.2.2. Particle Sizing Counter Analysis of the Immobilized Catalysts

The particle size of the immobilized catalysts was measured after blending the powders in distilled water under continuous stirring. The average particle size was determined using a laser particle counter PC-2200 (Spectrex Corp., Redwood, CA, USA) in a 150 mL sample chamber (0.1 g/L). Particle counting was operated with a laser diode at a wavelength of 650 nm.

#### 3.2.3. XRD Analysis of the Immobilized Catalysts

The phase composition of immobilized catalysts before and after use was studied by XRD analysis using a Rigaku SmartLab SE X-ray powder diffractometer with Cu Kα radiation (λ = 0.154 nm) for phase identification. Full pattern identification was carried out by a SmartLab Studio II software package, version 4.2.44.0 by Rigaku Corporation (Tokyo, Japan). Materials identification and analysis were performed by the ICDD base PDF-2 Release 2019 (Powder Diffraction File, ver. 2.1901). XRD patterns were obtained using 40 kV, 30mA by Θ/2Θ (Bragg-Brentano geometry) in the 2Θ range of 10–90° (step size 0.03° and speed 4°/min). The XRD measurements were performed for both freshly synthesized and used matrices, when the latter were separated from the reaction mixture and dried.

#### 3.2.4. X-ray Photoelectron Spectroscopy (XPS) Analysis of the Immobilized Catalysts

The phase composition of immobilized catalysts before and after the use was carried out by a NEXSA XPS system (Thermo Fisher Scientific, East Grinstead, UK) with a monochromatized Al Kα source (400 μm diameter).

### 3.3. Esterification of OA under Ultrasonic Activation in a Batch Mode

Esterification of OA was performed according to our own method [12], with modifications. In brief, 0.4 mL OA (99% Sigma, Burlington, MA, USA) was added to 8.1 mL methanol (Bio-Lab., Ashkelon, Israel), 5 mL n-hexane (Bio-Lab., Ashkelon, Israel) and 4.0 g of a catalyst powder (BSil, AlSil or BlankSil), in a sealable 50 mL bottle. The reaction bottle was placed in an ultrasonic bath (Elma, Germany; Elmasonic *p* 30 H) containing water for 15–60 min at ambient temperature. After the reaction, the bottles were cooled to ambient temperature in a water bath. After the reaction, an aliquot of water equal to the reaction mixture volume was added and the mixture was centrifuged for 2 min at 1500 rpm (Beckman Coulter, Brea, CA, USA) using a JA-25.50 rotor (Beckman Coulter). After the centrifugation, the two phases were separated, and the phase composition was analyzed using HPLC (Section 3.5).

### 3.4. Continuous Esterification of OA under Ultrasonic Activation in A Fixed-Bed Reactor

The esterification reaction was carried out in a continuous mode using the fixed-bed submerged in an ultrasonic bath, as shown in Figure 7. The reactor was designed with a Π-shaped tube made from borosilica glass with an internal diameter of 3.15 cm and an inner volume of 257 mL, filled with 150 g of matrix. For ultrasonic activation of the reaction, an ultrasonic bath (37 kHz, Elma, Germany; Elmasonic *p* 70 H) filled with distilled water at ambient temperature was used. A reaction mixture composed of OA (70% purity, Fisher Chemical, Hampton, NH, USA), methanol and hexane in a volume ratio of 1:20:120, respectively, was flowed into the reactor using a multichannel peristaltic pump ECOLINE VC-MS/CA8-6 (Ismatec, Wertheim, Germany) at a rate of 4.3 mL/min. At the outlet, the reaction mixture was withdrawn by the same pump at the same flow rate. The 5 mL samples were collected hourly and extracted with equal volumes of water. After centrifugal separation (Section 3.3), the phase composition was determined by HPLC (Section 3.5).

### 3.5. HPLC Analysis of the Samples

Samples of each of the phases were filtered twice using a 0.45 μm polytetrafluoroethylene filter (PTFE, Membrane solutions, Nantong, China) and analyzed by HPLC using the protocol we described earlier [12] using UHPLC (UltiMate, Dionex, Hamburg, Germany) equipped with a Corona Ultra RS detector (Thermo Scientific, Bremen, Germany) on a Sepax Poly PP-100, 5 μm, 4.6 × 250 mm column (Sepax Technologies, Inc., Newark, DE, USA) in an isocratic regime using 100% acetonitrile (Bio-Lab., Ashkelon, Israel) as the eluent. The column temperature was 30 °C, the flow rate was 1.2 mL/min, and the injection volume was 2 µL.

### 3.6. Statistical Analysis

The results were obtained from at least three independent experiments carried out in duplicate and analyzed by single-factor ANOVA analyses. Quantitative results are presented as the mean ± standard deviation (SD).

## 4. Conclusions

In conclusion, esterification of FFAs can be performed in ambient conditions using heterogeneous catalysts based on Lewis acids under ultrasonic activation. A 100% yield of biodiesel was achieved after 60 min using the BSil matrix catalyst. It was proven that the catalyst could be recycled several times. Incorporation of the Lewis acid catalysts into the sol–gel matrix allows their reuse and reapplication for continuous biodiesel production. Since FFAs are the major component of fatty waste from the food industry (brown grease), the latter can serve as a potential source for biodiesel production using the above-described method.

## 5. Patent

Nisnevitch M., Nakonechny F., Kolet M., Zerbib D. and Albo Y. Production of Biodiesel. US Provisional Patent Application No. 62/754,033.

## Figures and Tables

**Table 1:** Comparison between freshly prepared and used BSil characterized by X-ray photoelectron spectroscopy (XPS).

Atomic %	BSil New	BSil Used
B1s	8.97	6.45
Si2p	25.99	28.38
F1s	7.43	7.37
O1s	57.61	57.80
